# Relationship between domain-specific physical activity and cognitive function in older adults – findings from NHANES 2011–2014

**DOI:** 10.3389/fpubh.2024.1390511

**Published:** 2024-07-24

**Authors:** Sijun Wu, Lin Wang, Shijie Liu, Juancai Qi, Fengrui Shi, Huiqi Zhuang, Youling Qian, Linqi Mei, Maolin Zhang

**Affiliations:** ^1^School of Physical Education, Wuhan University of Technology, Wuhan, China; ^2^School of Physical Education, Hubei Minzu University, Enshi, China; ^3^School of Physical Education, Hubei University, Wuhan, China; ^4^School of Wushu, Shandong Sport University, Jinan, China

**Keywords:** physical activity, older adult, cognitive function, executive function, MET, NHANES

## Abstract

**Objective:**

To determine the relationship between domain-specific physical activity (PA) (e.g., occupational PA [OPA], transport-related PA [TPA], and recreational PA [RPA]) and cognitive function in older adults.

**Methods:**

The data was obtained from the 2011–2014 cycle of the NHANES. We utilized weighted multivariate linear regression models among the included 2,924 people aged 60 years or older for our purposes.

**Results:**

RPA and total PA according to WHO guidelines were associated with verbal fluency (RPA *β*: 1.400, 95% CI: 0.776, 2.024, *p* = 0.002; total PA *β*: 1.115, 95% CI: 0.571, 1.659, *p* = 0.001), processing speed and executive function (RPA *β*: 2.912, 95% CI. 1.291, 4.534, *p* = 0.005; total PA *β*: 2.974, 95% CI: 1.683, 4.265, *p* < 0.001) were positively correlated, and total PA was correlated with delayed memory performance (*β*: 0.254, 95% CI: 0.058, 0.449, *p* = 0.019). No significant association was observed between OPA, TPA, and various aspects of cognitive function among individuals over 60 years.

**Conclusion:**

There was no noteworthy correlation discovered between OPA and TPA in relation to cognitive function. However, RPA and total PA exhibited significant associations with verbal fluency, processing speed, and executive function. Additionally, maintaining PA levels ranging from 600 to 1,200 MET-min/week would yield the most favorable outcomes for cognitive function.

## 1 Introduction

Recent data indicates a rapid global increase in the older adult population, with an estimated 2.1 billion individuals aged 60 and above expected by the mid-21st century (1). Aging is often accompanied by a decrease in cognitive abilities, including memory, verbal expression, attention, and executive function (2). Without effective intervention, the age-related cognitive function decline can be a precursor to cognitive impairment related to dementia or Alzheimer’s disease (AD) (2). Currently, more than 55 million people are suffering from dementia, with AD being the most common form of cognitive decline in older individuals (3). In 2019, healthcare expenditures resulting from dementia reached $1.3 trillion, imposing a substantial financial burden on governments, families, and individuals (4). Due to the current lack of effective drugs for dementia worldwide, there is an urgent requirement for a cost-effective, non-pharmacological intervention that can help maintain and enhance cognitive function in older adults.

Physical activity (PA) has been shown to be beneficial in assisting in the treatment of illnesses like diabetes (5), depression (6), liver and gallbladder disease (7), cancer (8), chronic kidney disease (9). PA has also been shown to positively impact body composition by increasing muscle mass (10), reducing body fat percentage (11), and enhancing bone mineral density (12). Physical activity has become a kind of low cost benefits of effective non-pharmaceutical interventions (13, 14). It is important to note that PA covers a variety of intricate behaviors, classified into three primary domains by the Global Physical Activity Questionnaire (GPAQ): occupational PA (OPA), transport-related PA (TPA), and recreational PA (RPA) (15). OPA includes a range of tasks that necessitate completion, whether they involve remuneration or not, including academic or vocational pursuits, household chores, and tending to gardens; TPA pertains to the mode of transportation typically employed by individuals to commute to various destinations (workplace, educational institutions, shopping centers), encompassing activities like walking or cycling; and RPA is defined as a sport, fitness or leisure activity that is carried out in addition to the two physical activities listed above (16). Different domains of PA may have different effects: studies indicate that OPA and TPA are not effective in preventing or inhibiting the development of diabetes (5) and depression (6), while RPA has shown more favorable outcomes. A study has also indicated that OPA, but not TPA and RPA, is related to a lower risk of chronic kidney disease (9).

Cognitive functions encompass various aspects such as memory, attention, perception, and thinking, constituting higher brain functions (17). According to neurocognitive psychiatry textbooks, cognitive functions are categorized into 12 domains: visual–spatial abilities, attention, problem-solving, general intelligence, psychomotor speed, sensory processing, verbal memory, non-verbal memory, processing speed, motor control/performance, working memory, and verbal reasoning (18). Previous researches have mainly investigated the correlation between physical activity and cognitive function in older adults, particularly focusing on RPA and specific types of exercise (19–24). For example, one study discovered that RPA exhibited a positive correlation with cognitive function in older adults with shorter sleep durations (19), and that aerobic exercise [walking (24), dancing (23)], resistance exercise (22), and mind–body exercise [tai chi (20), yoga (21)] also had beneficial effects on cognitive function in older individuals. Nonetheless, engaging in these forms of exercise is not indicative of physical activity in a specific domain and is mostly done during leisure time for health benefits. Based on this, there is currently a lack of research exploring the association between specific domains of PA (OPA, TPA, and RPA) and cognitive function in older adults, and whether these associations might be influenced by sociodemographic factors (such as gender, age, race, and education) and behavioral characteristics (like smoking and alcohol consumption).

Therefore, we propose the following hypotheses:

*H1*: Different domains of PA (OPA, TPA, and RPA) may exhibit varying associations with cognitive function in older adults.

*H2*: The associations between different domains of physical activity and cognitive function in older adults may be influenced by sociodemographic factors (gender, age, race, education) and behavioral characteristics (smoking, alcohol consumption).

## 2 Methods

### 2.1 Study population

The purpose of the National Health and Nutrition Examination Survey (NHANES) is to evaluate the health and nutritional well-being of adults and children residing in the United States, while also ensuring that the sample represents the non-institutionalized civilian population of the country (25). Since all data collection procedures in NHANES were performed in compliance with the research ethics regulations of the National Center for Health Statistics Review Board, no additional ethical review was required for this study.

**Figure 1:**
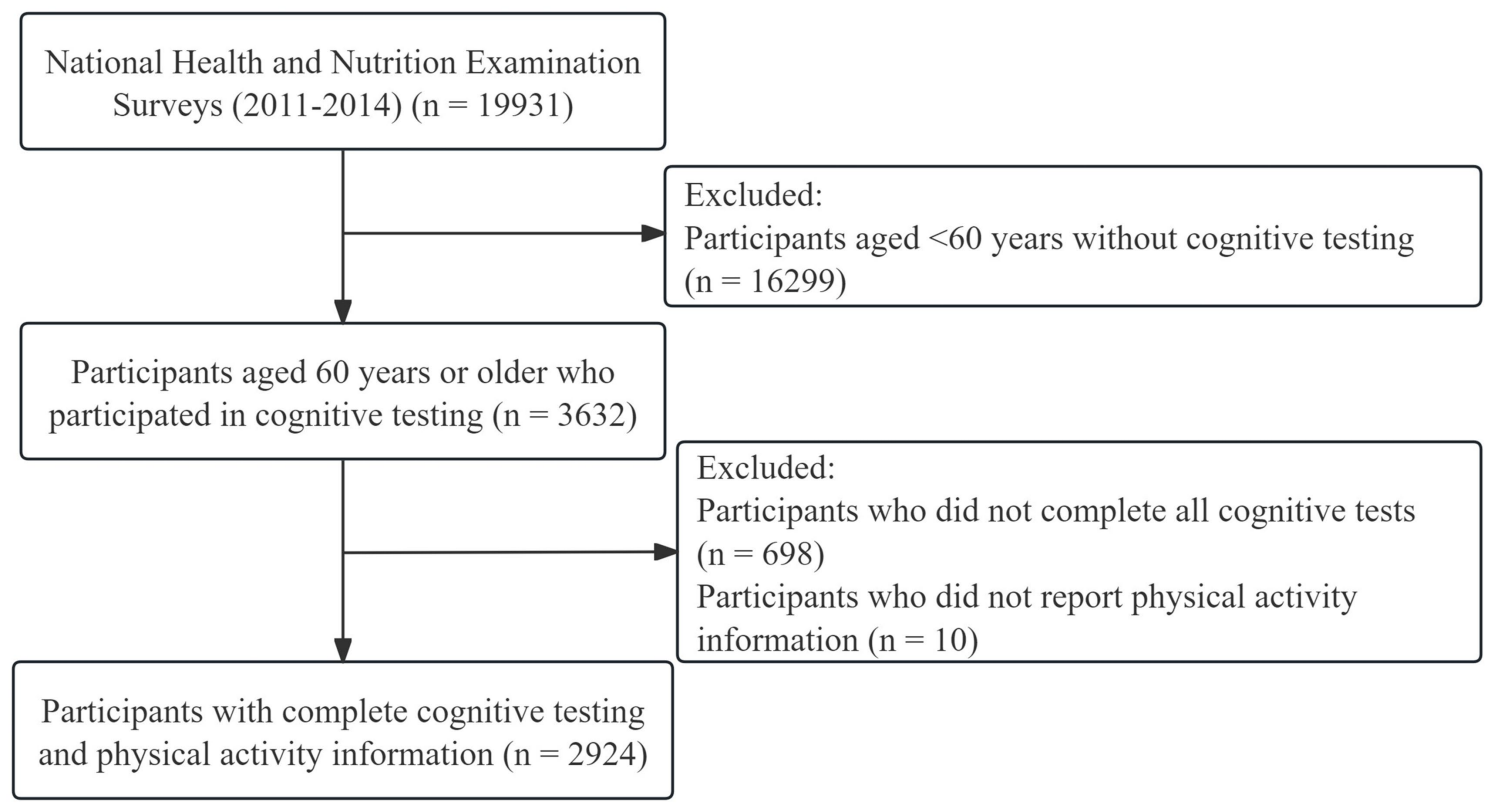
Flow chart of research sample selection.

In the survey conducted from 2011 to 2014, a total of 19,331 participants were initially involved. Given that the cognitive tests of interest in this study were specifically administered to individuals aged 60 and older, participants under the age of 60 were excluded from the analysis (*n* = 16,299). Subsequently, from the remaining 3,632 participants, those who were missing cognitive test data (*n* = 698) or information on physical activity (*n* = 10) were further excluded. Finally, 2,924 participants were included in the current analyses, and the screening process is shown in Figure 1.

### 2.2 Physical activity

The Global Physical Activity Questionnaire (GPAQ) was utilized to evaluate participants’ physical activity, which consisted of the frequency of physical activity (1–7 times per week), the duration of a single exercise session (minutes per session), and the intensity of the exercise (moderate- or vigorous-intensity) in a typical week for three different PA domains (OPA, TPA, and TPA) (26). PA in each domain is measured as the corresponding metabolic equivalent (MET) multiplied by the number of minutes of activity per week (MET-min/week). According to the MET score recommended by NHANES, moderate OPA, TPA, and RPA, which result in a slight increase in respiration or heart rate, were classified as 4 MET, whereas vigorous OPA and TPA, which lead to a significant increase in respiration or heart rate, were identified as 8 MET (16). In addition, the activity levels of OPA, TPA and RPA were pooled to obtain total PA.

As the WHO Physical Activity and Sedentary Behavior Guidelines (hereafter referred to as the guidelines) propose that individuals aged 60 and above should engage in a minimum of 150–300 min per week of moderate-intensity aerobic activity (equivalent to 600–1,200 MET-min/week) for considerable health benefits. Further advantages can be attained by exceeding 300 min per week (>1,200 MET-min/week) of moderate-intensity aerobic activity (27). Therefore, we first categorized the amount of physical activity in each domain into two groups, meeting guideline recommendations and not meeting guideline recommendations, to assess the effect on cognitive function of those meeting guideline recommendations compared to those not meeting guideline recommendations. The amount of physical activity in each domain was then further categorized into 4 groups (1) none (0 MET-min/week), (2) low (<600 MET-min/week), (3) moderate (600–1,200 MET-min/week), and (4) high (>1,200 MET-min/week) to assess the relationship between different doses of domain-specific PA and cognition.

### 2.3 Cognitive function

Trained interviewers assisted participants in a series of cognitive tests (28). The Consortium to Establish a Registry for Alzheimer’s Disease Word Learning Subtest (CERAD W-L) consists of three immediate recall (CERAD-IR, total score of 30) and one delayed recall (CERAD-DR, total score of 10) tests to assess immediate and delayed learning of new verbal information (29). The test has been shown to be effective in distinguishing the presence of underlying cognitive impairment (30, 31). For CERAD-IR, participants were instructed to orally read aloud 10 unrelated words one by one and then immediately recall as many words as possible after their presentation, which was repeated three times with different word orders each time. Delayed word recall followed the completion of the subsequent two cognitive test items (32). The number of words recalled correctly is the score for the test. The Animal Fluency test (AF) assesses categorical verbal fluency by requiring participants to name as many different animals as possible within 1 min, with one name per animal. This score distinguishes individuals with normal cognitive function from those with mild cognitive impairment and more severe forms of cognitive decline, such as Alzheimer’s disease (33, 34). The Digit Symbol Substitution test (DSST), a component of the Wechsler Adult Intelligence Scale (WAIS-III), comprehensively evaluates processing speed, sustained attention, and executive function (35). Administered on paper, the test sheet includes a key with 9 numbers and symbols. Participants have 2 min to transcribe the corresponding symbols into 133 boxes next to the numbers; the score reflects the total number of correct matches (36). Higher scores across all tests indicate better cognitive function.

### 2.4 Covariates

Based on previous studies (19, 37), we adjusted for several confounders that may influence cognitive function and physical activity. These confounders include demographics such as sex (male, female), age (60–69 years, 70–79 years, 80+ years), race, educational attainment, and poverty income ratio (PIR). Race was further divided into Non-Hispanic White, Non-Hispanic Black, Other Hispanic, Mexican American and Other/multiracial. Educational attainment was categorized from lowest to highest as Less Than 9th Grade, 9th-11th Grade, High School Grad/GED, Some College or AA degree, and College Graduate or above. The PIR is calculated based on household income in relation to the poverty guidelines set by the government, with higher values representing better household economic conditions. Body Mass Index (BMI) is obtained by calculating the ratio of weight in kilograms to the square of height in meters. Lifestyle factors were primarily assessed by evaluating participants’ smoking and drinking habits. Smoking status was determined through questions such as ‘Have you smoked at least 100 cigarettes?,’ ‘Do you currently smoke?,’ and ‘How long ago did you quit smoking?’ Participants were divided into three groups: ‘current smokers,’ ‘former smokers,’ and ‘never smokers.’ Drinking status was classified into four groups based on the frequency of drinking: ‘non-drinkers,’ ‘1–5 drinks per month,’ ‘5–10 drinks per month,’ and ‘10+ drinks per month.’ This classification was determined using criteria such as ‘drinking at least 12 times a year,’ ‘having imbibed at least 12 alcoholic beverages throughout their lifetime,’ and the frequency of drinking in the past 12 months (weekly, monthly, yearly). Additionally, taking into account the potential impact of the illness on cognitive performance and physical activities, we identified depression, diabetes, hypertension, and cardiovascular-related diseases by combining participants’ self-reports with objective measures. Depression was assessed using the Patient Health Questionnaire-9 (PHQ-9), which consists of 9 items worth a total of 27 points, with a total score of ≥10 considered to be depression (38). Diabetes was defined by fulfilling any of the subsequent requirements: (1) Being informed by a doctor about having diabetes, (2) Currently taking anti-diabetic drugs, or (3) Fasting blood glucose (mmol/l) ≥ 7.0. Hypertension was diagnosed as having an average systolic blood pressure ≥ 140 mmHg or diastolic blood pressure ≥ 90 mmHg, or if the subject reported taking prescription drugs for hypertension (39). Cardiovascular diseases were defined as having been reported to the subject by medical staff as congestive heart failure, coronary artery disease, angina pectoris, or heart disease. In addition, a ‘trouble remembering’ group was created based on the frequency of memory problems the subjects had experienced in the past 7 days: never, about once, two or three times, almost every day, and several times a day.

### 2.5 Statistical analysis

The NHANES study used a complex multi-stage probability sampling design, which requires specific weighting procedures during analysis. To obtain the weight variables needed for this study, we divided the weight of the two-cycle Mobile Examination Center interviews by 2, following the recommendations of Tutorials (32). Since deleting missing covariates directly would result in the loss of information on physical activity and cognitive function, and considering that there were only a few missing covariates (Supplementary Table 1), we used the MICE package in R software to impute the missing variables in order to avoid potential bias.

The baseline characteristics of the participants in the study were analyzed by presenting the means and standard deviations for continuous variables, as well as frequencies (%) for categorical variables. T-tests or chi-squared tests were used to determine any significant differences between the groups. Considering that the dependent variable of interest in this study is a continuous variable, the relationship between domain-specific PA and cognitive function was examined by calculating effect sizes (β) with 95% confidence intervals (CI) using weighted multivariate linear regression models. Three models were used: Model 1, which was not adjusted for any variables, and Model 2, which was adjusted for age, sex, PIR, race, BMI, educational attainment, smoking and alcohol consumption. Model 3 served as a fully adjusted model, further adjusting for depression, diabetes, hypertension, cardiovascular diseases, and difficulty with memory in the past 7 days based on model 2. In addition, the fully adjusted model was used to perform subgroup analyses, grouping people according to age, sex, race, educational attainment, smoking and drinking status, to ascertain the relationship between domain-specific PA and cognitive function in different subgroups. Finally, sensitivity analyses were performed by adding PA volumes from the other two domains to model 3, to avoid potential benefits of simultaneous PA in multiple domains. All statistical analyses were conducted using the R software (version 4.3.0) and *p* < 0.05 for two-tailed test indicates statistical significance.

## 3 Results

### 3.1 Descriptive characteristics

The 2,924 respondents included in this study (Table 1) represented a total of 53,109,681 US non-institutionalized civilian population, the majority of whom were non-Hispanic white (*n* = 1,396), with 1,504 (54.54%) females versus 1,420 (45. 46%) males, and an overall mean age of 69.20 (6.65) years, BMI 29.06 (6.29) kg/m^2^, PIR 3.12 (1.58), and cognitive test scores were CERAD-IR 19.72 (4.49), CERAD-DR 6.23 (2.30), AF 18.08 (5.70) and DSST 51.96 (16.81). Half of the respondents were non-smokers [1,443 (49.72%)] and consumed alcohol 1–5 times per month [1,379 (47.46%)]. Most were free of depression [2,598 (92.72%)], diabetes [2,106 (76.49%)] and cardiovascular disease [2,415 (82.40%)], except for hypertension [1,970 (63.61%)], which was more common. The percentages of people with OPA, TPA and RPA were 34, 19, and 45% respectively, and 33% were not physically active in any domain. In all domains, those who were physically active were younger than those who were not (*p* < 0.05), had higher AF test and DSST scores; with the exception of TPA, participants with OPA or RPA had better economic conditions and higher CERAD.IR and CERAD.DR scores compared to those without (*p* < 0.05).

**Table 1:** Characteristics of study population.

Characteristic	Overall, *N* = 2,924 (100%)^a^	OPA		TPA		RPA		Total PA	
		No, *N* = 2045 (66%)^a^	Yes, *N* = 879 (34%)^a^	No, *N* = 2,314 (81%)^a^	Yes, *N* = 610 (19%)^a^	No, *N* = 1706 (55%)^a^	Yes, *N* = 1,218 (45%)^a^	No, *N* = 1,031 (33%)^a^	Yes, *N* = 1893 (67%)^a^
Age^1,2,3,4^	69.20 (6.65)	69.78 (6.81)	68.11 (6.20)	69.56 (6.71)	67.67 (6.15)	69.74 (6.76)	68.55 (6.45)	70.46 (6.87)	68.57 (6.45)
Age (Group)^1,2,3,4^									
60–69 years	1,464 (51.72%)	969 (47.75%)	495 (59.29%)	1,095 (49.10%)	369 (62.92%)	816 (48.88%)	648 (55.21%)	442 (43.40%)	1,022 (55.91%)
70–79 years	806 (27.48%)	561 (27.05%)	245 (28.30%)	672 (28.99%)	134 (21.03%)	479 (28.38%)	327 (26.38%)	298 (28.88%)	508 (26.78%)
80+ years	654 (20.80%)	515 (25.20%)	139 (12.41%)	547 (21.91%)	107 (16.05%)	411 (22.74%)	243 (18.41%)	291 (27.73%)	363 (17.31%)
Sex^1,2,4^									
Female	1,504 (54.54%)	1,113 (57.50%)	391 (48.91%)	1,223 (56.29%)	281 (47.09%)	907 (56.55%)	597 (52.07%)	604 (61.02%)	900 (51.27%)
Male	1,420 (45.46%)	932 (42.50%)	488 (51.09%)	1,091 (43.71%)	329 (52.91%)	799 (43.45%)	621 (47.93%)	427 (38.98%)	993 (48.73%)
PIR^1,3,4^	3.12 (1.58)	3.01 (1.60)	3.32 (1.52)	3.11 (1.58)	3.16 (1.60)	2.85 (1.58)	3.44 (1.52)	2.77 (1.58)	3.29 (1.55)
Race^1,2,3^									
Non-Hispanic White	1,396 (79.48%)	924 (76.76%)	472 (84.67%)	1,170 (80.68%)	226 (74.35%)	820 (78.68%)	576 (80.46%)	510 (78.70%)	886 (79.87%)
Non-Hispanic Black	697 (8.44%)	502 (9.41%)	195 (6.60%)	549 (8.42%)	148 (8.53%)	417 (9.14%)	280 (7.59%)	254 (9.43%)	443 (7.94%)
Other Hispanic	294 (3.66%)	223 (4.26%)	71 (2.50%)	191 (3.02%)	103 (6.37%)	185 (4.22%)	109 (2.97%)	92 (3.62%)	202 (3.67%)
Other/multiracial	281 (5.04%)	216 (5.93%)	65 (3.34%)	200 (4.57%)	81 (7.02%)	132 (4.28%)	149 (5.97%)	76 (4.27%)	205 (5.42%)
Mexican American	256 (3.39%)	180 (3.64%)	76 (2.90%)	204 (3.31%)	52 (3.73%)	152 (3.69%)	104 (3.02%)	99 (3.97%)	157 (3.09%)
Education attainment^1,2,3,4^									
Less Than 9th Grade	329 (5.70%)	255 (6.97%)	74 (3.28%)	252 (5.74%)	77 (5.56%)	236 (7.96%)	93 (2.94%)	147 (9.06%)	182 (4.01%)
9-11th Grade	415 (10.27%)	313 (11.43%)	102 (8.07%)	316 (10.27%)	99 (10.28%)	300 (13.64%)	115 (6.15%)	178 (13.43%)	237 (8.68%)
High School Grad/GED	684 (22.19%)	475 (22.21%)	209 (22.15%)	570 (23.74%)	114 (15.56%)	425 (24.32%)	259 (19.57%)	266 (25.88%)	418 (20.33%)
Some College or AA degree	821 (31.29%)	523 (27.90%)	298 (37.75%)	649 (31.06%)	172 (32.25%)	460 (32.48%)	361 (29.83%)	252 (28.04%)	569 (32.93%)
College Graduate or above	672 (30.55%)	476 (31.49%)	196 (28.75%)	526 (29.19%)	146 (36.35%)	283 (21.61%)	389 (41.52%)	188 (23.60%)	484 (34.05%)
Drinking status^3,4^									
Non-drinker	916 (27.35%)	675 (28.81%)	241 (24.59%)	730 (28.08%)	186 (24.24%)	564 (31.97%)	352 (21.71%)	358 (33.08%)	558 (24.49%)
1–5 drinks/month	1,379 (47.46%)	949 (47.27%)	430 (47.84%)	1,101 (47.65%)	278 (46.67%)	822 (48.84%)	557 (45.79%)	490 (49.07%)	889 (46.66%)
5–10 drinks/month	122 (5.00%)	80 (4.20%)	42 (6.50%)	91 (4.28%)	31 (8.03%)	61 (3.87%)	61 (6.37%)	26 (2.43%)	96 (6.28%)
10+ drinks/month	452 (20.19%)	301 (19.72%)	151 (21.08%)	346 (19.98%)	106 (21.06%)	218 (15.33%)	234 (26.13%)	130 (15.42%)	322 (22.57%)
Smoking status^3^									
Current smoker	371 (10.96%)	254 (10.61%)	117 (11.61%)	277 (10.63%)	94 (12.36%)	269 (14.69%)	102 (6.37%)	143 (13.03%)	228 (9.91%)
Former smoker	1,108 (39.33%)	760 (37.91%)	348 (42.02%)	898 (39.67%)	210 (37.86%)	626 (36.87%)	482 (42.35%)	380 (36.47%)	728 (40.77%)
Never smoker	1,443 (49.72%)	1,030 (51.47%)	413 (46.36%)	1,138 (49.70%)	305 (49.77%)	811 (48.44%)	632 (51.28%)	508 (50.50%)	935 (49.32%)
BMI^2,3,4^	29.06 (6.29)	29.28 (6.60)	28.65 (5.64)	29.31 (6.40)	28.00 (5.70)	29.95 (6.78)	27.99 (5.46)	30.48 (7.20)	28.37 (5.67)
Trouble remembering^3,4^									
No	1,626 (54.52%)	1,152 (55.39%)	474 (52.89%)	1,280 (54.79%)	346 (53.41%)	955 (55.41%)	671 (53.43%)	565 (55.10%)	1,061 (54.24%)
About once	693 (26.43%)	460 (25.73%)	233 (27.76%)	541 (26.18%)	152 (27.49%)	377 (23.95%)	316 (29.47%)	217 (23.58%)	476 (27.86%)
Two or three times	435 (14.06%)	304 (13.83%)	131 (14.50%)	356 (14.27%)	79 (13.18%)	252 (14.33%)	183 (13.72%)	163 (14.32%)	272 (13.93%)
Nearly every day	116 (3.57%)	85 (3.31%)	31 (4.06%)	93 (3.30%)	23 (4.72%)	81 (4.22%)	35 (2.77%)	56 (4.48%)	60 (3.11%)
Several times a day	50 (1.42%)	40 (1.75%)	10 (0.80%)	41 (1.48%)	9 (1.20%)	38 (2.09%)	12 (0.61%)	27 (2.53%)	23 (0.87%)
Depression^3,4^									
No	2,598 (92.72%)	1,801 (91.96%)	797 (94.15%)	2,046 (92.14%)	552 (95.16%)	1,463 (90.16%)	1,135 (95.83%)	868 (88.74%)	1,730 (94.69%)
Yes	261 (7.28%)	196 (8.04%)	65 (5.85%)	214 (7.86%)	47 (4.84%)	195 (9.84%)	66 (4.17%)	132 (11.26%)	129 (5.31%)
Hypertension^2,3,4^									
No	954 (36.39%)	640 (34.94%)	314 (39.16%)	706 (34.72%)	248 (43.51%)	513 (32.29%)	441 (41.42%)	277 (29.30%)	677 (39.96%)
Yes	1,970 (63.61%)	1,405 (65.06%)	565 (60.84%)	1,608 (65.28%)	362 (56.49%)	1,193 (67.71%)	777 (58.58%)	754 (70.70%)	1,216 (60.04%)
Diabetes^1,2,3,4^									
No	2,106 (76.49%)	1,442 (74.36%)	664 (80.55%)	1,643 (75.50%)	463 (80.74%)	1,203 (73.43%)	903 (80.24%)	697 (70.71%)	1,409 (79.40%)
Yes	817 (23.51%)	602 (25.64%)	215 (19.45%)	670 (24.50%)	147 (19.26%)	502 (26.57%)	315 (19.76%)	333 (29.29%)	484 (20.60%)
Cardiovascular disease^2,3,4^									
No	2,415 (82.40%)	1,702 (83.37%)	713 (80.54%)	1,871 (80.71%)	544 (89.61%)	1,369 (80.21%)	1,046 (85.09%)	815 (78.80%)	1,600 (84.21%)
Yes	508 (17.60%)	343 (16.63%)	165 (19.46%)	442 (19.29%)	66 (10.39%)	336 (19.79%)	172 (14.91%)	216 (21.20%)	292 (15.79%)
CERAD.IR^1,3,4^	19.72 (4.49)	19.47 (4.63)	20.20 (4.17)	19.65 (4.47)	20.03 (4.58)	19.20 (4.58)	20.36 (4.30)	18.97 (4.74)	20.10 (4.31)
CERAD.DR^1,3,4^	6.23 (2.30)	6.10 (2.40)	6.48 (2.09)	6.18 (2.32)	6.43 (2.20)	6.00 (2.34)	6.51 (2.22)	5.84 (2.45)	6.43 (2.20)
Animal Fluency^1,2,3,4^	18.08 (5.70)	17.62 (5.87)	18.95 (5.25)	17.86 (5.66)	19.00 (5.76)	16.98 (5.45)	19.44 (5.70)	16.37 (5.30)	18.94 (5.70)
DSST^1,2,3,4^	51.96 (16.81)	50.38 (17.54)	54.98 (14.86)	51.57 (16.84)	53.62 (16.61)	48.47 (17.10)	56.24 (15.40)	46.67 (17.35)	54.63 (15.88)

*t*-test adapted to complex survey samples and chi-squared test with Rao & Scott’s second-order correction were used to calculate between-group differences.

^1,2,3,4^Represents the within-group differences of variables in people with and without OPA, TPA, RPA or total PA, respectively.

a Mean (SD) for continuous; *n* (%) for categorical.

### 3.2 Relationship between domain-specific PA and cognitive function

Table 2 presents the results of the weighted multivariate linear regression model. In model 3 (fully adjusted), OPA, TPA, and RPA that met guideline recommendations were not significantly associated with immediate versus delayed recall compared to those who did not meet the guidelines. However, total PA showed a significant association with better delayed recall performance (*β*: 0.254, 95% CI: 0.058, 0.449, *p* = 0.019). For the AF test, adherence to guideline recommendations for OPA (*β*: 0.011, 95% CI: −0.461, 0.483, *p* = 0.955) and TPA (*β*: 0.179, 95% CI: −0.955, 1.312, *p* = 0. 713) did not show a significant association, whereas RPA (*β*: 1.400, 95% CI: 0.776, 2.024, *p* = 0.002) and total PA (*β*: 1.115, 95% CI: 0.571, 1.659, *p* = 0.001) showed a significant association. Similarly, in DSST, meeting guideline recommendations for OPA (*β*: 0.821, 95% CI: −0.341, 5.272, *p* = 0.365) and TPA (*β*: −1.575, 95% CI: −3.596, 0.445, *p* = 0.105) remained non-significantly associated with test performance, whereas RPA (*β*: 2.912, 95% CI: 1.291, 4.534, *p* = 0.005) and total PA (*β*: 2.974, 95% CI: 1.683, 4.265, *p* < 0.001) were significantly associated with higher test scores.

**Table 2:** The relationship between Domain-specific PA and Cognitive function when meeting PA guidelines.

	Model 1		Model 2		Model 3	
	*β* (95% CI)	*p*-value	*β* (95% CI)	*p*-value	*β* (95% CI)	*p*-value
CERAD.IR	Reference^1^		Reference^1^		Reference^1^	
OPA^2^	0.575 (−0.009, 1.160)	0.053	0.127 (−0.322, 0.577)	0.554	0.133 (−0.371, 0.637)	0.542
TPA^2^	0.239 (−0.440, 0.917)	0.478	0.005 (−0.586, 0.595)	0.987	0.006 (−0.669, 0.682)	0.983
RPA^2^	**0.875 (0.445, 1.304)**	**<0.001**	0.208 (−0.198, 0.613)	0.291	0.131 (−0.337, 0.599)	0.519
Total PA^2^	**0.992 (0.661, 1.324)**	**<0.001**	**0.444 (0.122, 0.765)**	**0.010**	0.370 (−0.026, 0.766)	0.062
CERAD.DR	Reference^1^		Reference^1^		Reference^1^	
OPA^2^	**0.264 (0.042, 0.485)**	**0.021**	0.049 (−0.174, 0.272)	0.647	0.047 (−0.193, 0.287)	0.650
TPA^2^	0.188 (−0.191, 0.567)	0.320	0.042 (−0.304, 0.388)	0.799	0.034 (−0.351, 0.420)	0.834
RPA^2^	**0.499 (0.281, 0.718)**	**<0.001**	**0.255 (0.035, 0.474)**	**0.026**	0.216 (−0.041, 0.473)	0.085
Total PA^2^	**0.525 (0.361, 0.689)**	**<0.001**	**0.292 (0.118, 0.466)**	**0.003**	**0.254 (0.058, 0.449)**	**0.019**
Animal fluency	Reference^1^		Reference^1^		Reference^1^	
OPA^2^	**0.923 (0.280, 1.566)**	**0.006**	0.084 (−0.385, 0.552)	0.709	0.011 (−0.461, 0.483)	0.955
TPA^2^	1.025 (−0.066, 2.117)	0.065	0.438 (−0.547, 1.424)	0.360	0.179 (−0.955, 1.312)	0.713
RPA^2^	**2.701 (2.047, 3.356)**	**<0.001**	**1.509 (0.961, 2.057)**	**<0.001**	**1.400 (0.776, 2.024)**	**0.002**
Total PA^2^	**2.267 (1.702, 2.831)**	**<0.001**	**1.222 (0.702, 1.742)**	**<0.001**	**1.115 (0.571, 1.659)**	**0.001**
DSST	Reference^1^		Reference^1^		Reference^1^	
OPA^2^	**3.766 (1.229, 6.303)**	**0.005**	0.843 (−0.923, 2.609)	0.327	0.821 (−1.228, 2.869)	0.365
TPA^2^	0.715 (−1.733, 3.163)	0.556	−0.898 (−2.573, 0.777)	0.272	−1.575 (−3.596, 0.445)	0.105
RPA^2^	**7.593 (5.603, 9.582)**	**<0.001**	**3.247 (1.760, 4.735)**	**<0.001**	**2.912 (1.291, 4.534)**	**0.005**
Total PA^2^	**7.098 (5.521, 8.675)**	**<0.001**	**3.346 (2.136, 4.555)**	**<0.001**	**2.974 (1.683, 4.265)**	**<0.001**

Model 1 does not adjust any variables; model 2 adjusts according to age, sex, PIR, race, educational attainment, smoking and drinking; model 3 continues to adjust depression, diabetes, hypertension, cardiovascular disease and trouble remembering on the basis of model 2.

1 Reference corresponds to the groups in each domain of PA (OPA, TPA, RPA) and total PA that do not meet the PA guidelines.

2 Groups that correspond to PA (OPA, TPA, RPA) in each domain and total PA that meet the PA guidelines.Significant values (*p* < 0.05) are in bold.

Continuing with the subgroup analyses based on the fully adjusted model (Supplementary Table 2), it can be seen that adherence to recommended PA guidelines interacts with age, gender, race, education level, smoking, and alcohol consumption in influencing the relationship between specific domain PA and cognitive function (*p* < 0.05). Specifically, among older adults, both RPA and total PA were significantly associated with immediate recall, delayed recall, language fluency, processing speed, and executive function, while OPA enhanced immediate and delayed recall. Total PA positively correlated with cognitive test scores among non-Hispanic White individuals. RPA was significantly associated with higher AF test and DSST scores. RPA and total PA were linked to better AF test and DSST performance in subgroups characterized by alcohol consumption and smoking, whereas immediate and delayed recall were associated with individuals who did not smoke or consume alcohol. Total PA was associated with better delayed recall among women, whereas RPA correlated positively with processing speed and executive function in men. Additionally, education level did not affect associations between PA in various domains and CERAD.DR test performance; however, certain stratifications influenced associations between RPA, TPA, and CERAD.IR, AF test, and DSST.

### 3.3 Dose-effect analysis

Table 3 shows the dose–response relationship between domain-specific PA and cognitive function based on the fully adjusted model, and Supplementary Table 2 contains details of model 1 and model 2. Compared to the physically inactive population, no dose of OPA, TPA or RPA was significantly associated with scores on the CERAD W-L test, and only total PA (*β*: 0.321, 95% CI: 0.037, 0.604, *p* = 0.033) was associated with better delayed recall in the high activity group. In the AF test, OPA and TPA were not significantly correlated with test scores in all dose groups, whereas a significant and positive correlation was observed between RPA and scores in the group undergoing moderate activity (*β*: 1.313, 95% CI: 0.280, 2.346, *p* = 0.024) and the high activity (*β*: 1.726, 95% CI: 0.792, 2.659, *p* = 0.007). Total PA also showed a significant positive association with this cognitive score at all doses (*p* < 0.05). In the DSST, neither OPA nor TPA had a significant association with test scores, regardless of the dosage. However, RPA and total PA demonstrated a positive correlation with higher scores. It is noteworthy that individuals engaging in a total PA of 600–1,200 MET-min/week show higher effect sizes (*β*) in the relationship between AF tests and DSST scores compared to other activity levels. This suggests a significant association between achieving 600–1,200 MET-min/week of PA and better cognitive performance. Similarly, a comparable relationship exists between RPA and DSST.

**Table 3:** Dose–response relationships between domain-specific PA and cognitive function.

	CERAD.IR		CERAD.DR		Animal fluency		DSST	
	*β* (95% CI)	*p*-value	*β* (95% CI)	*p*-value	*β* (95% CI)	*p*-value	*β* (95% CI)	*p*-value
**OPA**								
None	reference		reference		reference		reference	
Low	0.432 (−0.264, 1.128)	0.160	0.323 (−0.120, 0.766)	0.113	1.165 (−0.157, 2.487)	0.071	2.466 (−0.341, 5.272)	0.071
Moderate	0.633 (−0.114, 1.379)	0.078	0.277 (−0.304, 0.857)	0.256	0.099 (−0.812, 1.010)	0.778	2.286 (−1.167, 5.739)	0.140
High	0.065 (−0.632, 0.761)	0.810	0.039 (−0.283, 0.361)	0.753	0.210 (−0.619, 1.039)	0.520	0.843 (−1.722, 3.407)	0.413
	*p* for trend^1^	0.958	*p* for trend	0.985	*p* for trend	0.756	*p* for trend	0.558
**TPA**								
None	reference		reference		reference		reference	
Low	0.246 (−0.670, 1.162)	0.497	0.167 (−0.326, 0.660)	0.400	0.392 (−0.642, 1.426)	0.352	2.122 (−0.559, 4.804)	0.093
Moderate	−0.015 (−0.915, 0.884)	0.964	0.047 (−0.598, 0.693)	0.848	0.774 (−0.972, 2.520)	0.286	−0.828 (−4.189, 2.534)	0.532
High	0.082 (−1.020, 1.185)	0.846	0.054 (−0.601, 0.708)	0.831	−0.446 (−1.676, 0.783)	0.370	−2.014 (−5.193, 1.165)	0.153
	p for trend	0.855	p for trend	0.771	p for trend	0.744	p for trend	0.116
**RPA**								
None	reference		reference		reference		reference	
Low	0.518 (−0.314, 1.349)	0.159	0.096 (−0.348, 0.541)	0.579	0.490 (−0.329, 1.309)	0.172	**2.120 (0.107, 4.132)**	**0.043**
Moderate	0.351 (−0.457, 1.160)	0.294	0.253 (−0.176, 0.683)	0.177	**1.313 (0.280, 2.346)**	**0.024**	**3.635 (1.363, 5.907)**	**0.011**
High	0.170 (−0.330, 0.671)	0.398	0.229 (−0.119, 0.577)	0.141	**1.726 (0.792, 2.659)**	**0.007**	**3.248 (0.564, 5.933)**	**0.028**
	*p* for trend	0.455	*p* for trend	0.090	*p* for trend	**<0.001**	*p* for trend	**0.010**
**Total PA**								
None	reference		reference		reference		reference	
Low	0.211 (−0.595, 1.017)	0.508	0.145 (−0.306, 0.595)	0.461	**0.987 (0.263, 1.711)**	**0.016**	**2.460 (0.473, 4.447)**	**0.023**
Moderate	0.512 (−0.213, 1.238)	0.121	0.257 (−0.064, 0.578)	0.098	**1.528 (0.459, 2.598)**	**0.013**	**4.720 (2.518, 6.922)**	**0.002**
High	0.414 (−0.060, 0.887)	0.076	**0.321 (0.037, 0.604)**	**0.033**	**1.412 (0.704, 2.119)**	**0.003**	**3.402 (1.758, 5.047)**	**0.002**
	*p* for trend	0.114	*p* for trend	**0.037**	*p* for trend	**<0.001**	*p* for trend	**0.005**

*β* and 95% CI are calculated based on the fully adjusted model. The results of Model 1 and Model 2 are shown in Supplementary Table 3.

1 *p* for trend: trends between the groups were examined by using the median of the PA volumes in each group as a continuous variable.Significant values (*p* < 0.05) are in bold.

### 3.4 Sensitivity analysis

Sensitivity analysis revealed (Supplementary Table 4) that OPA and TPA are still not significantly related to the cognitive function of older adults when the activity levels of the other two PA domains are added to the model 3. Except that the relationship between RPA and DSST score weakened in the low PA group (*β*: 1.960, 95% CI: −0.299, 4.220, *p* = 0.070), the other associations were still significant.

## 4 Discussion

This study presents, for the first time, varying associations between different domains of PA (OPA, TPA, RPA) and cognitive function in older adults. Regarding language fluency (AF) and processing speed, executive function (DSST), adherence to guideline-recommended OPA and TPA did not exhibit significant associations, whereas RPA showed significant correlations with total PA, supporting hypothesis H1.

We posit that RPA may have a stronger effect than OPA and TPA in adults aged 60 and above due to a potentially passive selection of OPA and TPA by older adults (40). Previous research has suggested that this phenomenon could be influenced by the nature of physical activity and psychological factors (41). Work-related physical activities, such as household chores, for older adults may be obligatory, accompanied by greater stress and discomfort (42). Another study indicated a 2.28-fold increase in stress levels following moderate to intense occupational PA (43). Prolonged exposure to stress triggers dysregulation of the hypothalamic–pituitary–adrenal (HPA) axis, leading to elevated glucocorticoid levels, which are neurotoxic to the prefrontal cortex and hippocampus, thereby affecting their normal functional expression including executive function and episodic memory (44). Furthermore, stress may impair cognitive function through behaviors such as smoking and alcohol consumption, recognized risk factors for cognitive impairment (45, 46). While these findings contrast with previous studies that found no associations between isolated RPA, OPA, TPA, and cognitive function (47), we attribute this discrepancy to differences in participant age ranges—prior studies included individuals aged 21–60, unlike our study’s focus on those aged 60 and above—and variations in cognitive assessment tools. Another reason we believe RPA yields superior effects over OPA and TPA is its social engagement aspect as a form of exercise, fitness, or recreational leisure activity (48). Research suggests that older adults with stronger social ties are less susceptible to cognitive decline (49). Studies such as NIE’s longitudinal investigation into the relationship between older adults’ social networks and cognitive function found a positive correlation between cognitive function and engagement in social activities and the size of one’s social circle; social gatherings and interactions provide emotional support (49). MOGIC’s systematic review, employing cross-sectional and cohort analyses, indicated that older adults receiving greater emotional support demonstrate better cognitive abilities. This viewpoint aptly explains why guideline-recommended OPA and TPA did not show significant associations (50). Furthermore, it’s notable that while RPA correlates with better language fluency, processing speed, and executive function, it shows no association with memory performance. This disparity may stem from different exercises selectively modulating specific brain regions to enhance diverse cognitive functions (51). For instance, resistance training has been shown to induce functional changes in the frontal lobe, thereby enhancing executive function (52). Conversely, studies have observed reduced gray matter in the hippocampus (linked to memory function) even after a year of stretching exercises (53).

Consistent with previous studies, neither TPA nor OPA showed significant associations with cognitive functions (54). Lund et al.’s prospective cohort study highlighted that physical exposure in occupational settings correlates with long-term sick leave, thereby compromising physical health to some extent (55). Additionally, it has been suggested that traffic-related air pollution may alter neurobehavioral functions (56). Considering environmental factors, studies have indicated that built environment and community characteristics influence TPA (57). Therefore, future research should explore how addressing neighborhood and architectural differences could promote PA adoption and alleviate disparities in cognitive function among older adults. Regarding TPA, while studies have suggested that activities like walking and biking effectively maintain muscle function and reduce disease risks (58, 59), our interpretation from this study’s findings is that cognitive functions involve higher-order brain activities, and independent TPA alone may not sufficiently impact cognitive function in older adults. Further research is needed to understand the varying associations between age-related TPA intensity and specific cognitive domains.

We also found that engaging in PA, regardless of whether it meets recommended guidelines, correlates with better AF test and DSST performance in older adults. This aligns with existing views that regular PA can improve overall cognitive function in older adults (60–62). This phenomenon may stem from the additional benefits of combining different types of exercises. Early meta-analyses suggest that combining aerobic and resistance training maximizes cognitive improvements in older adults (63), and multifaceted physical exercise has positive effects on overall cognition in MCI or dementia patients, particularly aerobic exercises (61). Notably, besides significantly correlating with AF test and DSST scores across all dosage groups, total PA is also associated with better delayed recall (CERAD-DR) in the high-activity group. Potential physiological mechanisms include frontal lobe gray matter volume reduction leading to decreased executive function (64), and hippocampal gray matter volume reduction closely tied to memory decline (65). Adequate physical activity has been shown to counteract or delay brain tissue atrophy due to aging and improve memory (22, 53, 66).

Furthermore, another finding of this study supports hypothesis H2. In subgroup analyses, we observed that age, race, gender, education level, smoking, and alcohol consumption can influence the relationship between different types of PA and cognitive function in older adults. Among demographic factors, older age groups showed significant correlations between regular physical activity (RPA) and total PA with immediate and delayed recall, language fluency, processing speed, and executive function, which is consistent with the findings of most previous studies (67, 68). A meta-analysis in China demonstrated that with every 5-year increase in age among adults aged 60 and above, the prevalence of mild cognitive impairment rises by 1.27–1.45 times, associated with degeneration in temporal and frontal brain structures due to accumulated DNA damage during brain aging, significantly impairing brain functions (69). Adequate RPA may therefore help to mitigate cognitive declines in older adults (70). Moreover, we found significant gender differences favoring males in delayed recall, language fluency, processing speed, and executive function. Although females experience faster cognitive decline than males due to changing estrogen levels during menopause, gender differences in various dimensions of cognitive function may be associated with higher levels of physical activity in older men, while postmenopausal type II muscle fiber loss leads to weakened muscle contraction in women, explaining why a higher proportion of older women transition to low physical activity compared to older men (71). Similarly, we found that education also influenced the associations between RPA and total PA with immediate recall, verbal fluency, processing speed, and executive function. A previous study reasonably interpreted the reason why higher education levels are likely to reduce the possibility of better cognitive function associated with physical activity and older adults with higher education levels are more likely to understand health care and brain training (47). Actively stimulating older adult learning can make up for the brain damage caused by aging, improve brain utilization efficiency, and maintain better cognitive function. It is worth noting that non-Spanish-speaking white people have better language fluency, processing speed, and executive function with RPA and total PA. In behavioral characteristics, the RPA and total PA of older adults who smoke and drink alcohol have differences in language fluency, processing speed, and executive function. The reason for this is that older adults who smoke and drink alcohol have degraded cognitive function, and daily alcohol consumption and monthly alcohol consumption days will lead to damage to neurons and the loss of synaptic connections, thereby affecting cognitive function (72, 73). We further clarified the dose of alcohol and found that older adults consuming 1–5 drinks/month exhibited more significant differences compared to those consuming 5–10 drinks/month. Previous survey studies also supported this view, showing that older adults who drink have a lower likelihood of cognitive impairment than non-drinkers. Moderate alcohol consumption has a protective effect on cognitive function, whereas excessive or no alcohol consumption does not provide this protection (74).

Finally, we found that meeting the recommended activity levels (600–1,200 MET-min/week) was associated with better AF and DSST scores in older adults for both regular physical activity (RPA) and total physical activity (TPA), while occupational physical activity (OPA) and transport-related physical activity (TPA) showed no significant correlation with cognitive test scores among this population group. This finding aligns with previous research suggesting that excessive exercise can lead to physical and mental fatigue, characterized by reduced activity in the prefrontal cortex, a crucial area for cognitive control (75). Our study also supports WHO guidelines on physical activity and sedentary behavior, indicating that meeting the minimum weekly total physical activity (>600 MET*minutes per week) is associated with enhanced cognitive function in adults aged 60 and above. While increasing physical activity further may still provide benefits, the range of 600–1,200 MET-min/week appears to offer optimal cognitive benefits.

There are some limitations to our study: first, owing to the cross-sectional design employed, the establishment of a causative connection between PA and cognitive performance was unattainable, for example, older adults with impaired cognitive abilities may not be physically active; second, physical activity was estimated by the amount of physical activity recalled by respondents during a typical week, which may introduce some bias, and at the same time, different types of exercise, intensity, frequency, and duration combinations are difficult to determine, which may result in different health benefits; third, although covariates were controlled for in the model, physical and mental health or physical activity in old age may still be affected by various other confounding factors, such as lack of space to exercise and environmental pollution. Fourth, although the association between domain-specific PA and cognitive function varied across subgroups, the results need to be interpreted with caution given the influence of the potential factors mentioned above. Finally, as the participants’ daily nutritional intake is complex and whichever nutrient is included in the analysis is somewhat biased, nutrition was not considered in this study, which presents some limitations.

## 5 Conclusion

OPA and TPA were not associated with all aspects of cognitive function in older adults over 60, and RPA and total PA were only positively correlated with some cognitive functions (verbal fluency, processing speed and executive function), total PA also demonstrates an association with delayed recall. In addition, achieving 600–1,200 MET*minutes per week of RPA and total PA may be associated with better processing speed and executive function in older adults. Further, rigorously designed randomized controlled trials and longitudinal studies are still necessary to validate our findings.

## Data availability statement

The publicly available data used in this study can be found at https://www.cdc.gov/nchs/nhanes/index.htm.

## Ethics statement

The studies involving humans were approved by NCHS Ethics Review Board (ERB) approval. The studies were conducted in accordance with the local legislation and institutional requirements. Written informed consent for participation was not required from the participants or the participants’ legal guardians/next of kin in accordance with the national legislation and institutional requirements.

## Author contributions

SW: Conceptualization, Formal analysis, Methodology, Software, Writing – original draft, Writing – review & editing. LW: Conceptualization, Data curation, Funding acquisition, Supervision, Writing – review & editing. SL: Data curation, Formal analysis, Supervision, Writing – review & editing. JQ: Data curation, Formal analysis, Supervision, Writing – review & editing. FS: Data curation, Formal analysis, Methodology, Supervision, Writing – review & editing. HZ: Data curation, Formal analysis, Supervision, Writing – review & editing. YQ: Data curation, Formal analysis, Supervision, Writing – review & editing. LM: Data curation, Formal analysis, Supervision, Writing – review & editing. MZ: Data curation, Formal analysis, Supervision, Writing – review & editing.
