# Supplementary material for: Relationship between domain-specific physical activity and cognitive function in older adults – findings from NHANES 2011–2014
Source: Front Public Health. 2024 Jul 24;12:1390511. doi: 10.3389/fpubh.2024.1390511 (PMC11303168; doi:10.3389/fpubh.2024.1390511)
Supplement: Supplementary file 1 [file Table_1.DOCX]

Contens

[Supplementary Table 1. Characteristics of study population before imputation 1](#_Toc169901851)

[Supplementary Table 2. Subgroup analysis 6](#_Toc169901852)

[Supplementary Table 2.1. CERAD.IR 6](#_Toc169901853)

[Supplementary Table 2.2. CERAD.DR 7](#_Toc169901854)

[Supplementary Table 2.3. Animal Fluency test（AF） 8](#_Toc169901855)

[Supplementary Table 2.4. Digit Symbol Substitution test (DSST) 9](#_Toc169901856)

[Supplementary Table 3. Dose-response relationships between domain-specific PA and cognitive function 11](#_Toc169901857)

[Supplementary Table 3.1. Dose-response relationships between domain-specific PA and cognitive function (based on model 1) 11](#_Toc169901858)

[Supplementary Table 3.2. Dose-response relationships between domain-specific PA and cognitive function (based on model 2) 12](#_Toc169901859)

[Supplementary Table 4. Sensitivity analysis 13](#_Toc169901860)

Supplementary Table 1. Characteristics of study population before imputation

| Characteristic | Overall, N =53,109,681  (100%)^a^ | OPA | | TPA | | RPA | | Total PA | |
| --- | --- | --- | --- | --- | --- | --- | --- | --- | --- |
|  |  | No, N = 34,831,426 (66%)^a^ | Yes, N = 18,278,255 (34%) ^a^ | No, N = 43,020,435 (81%) ^a^ | Yes, N = 10,089,246 (19%) ^a^ | No, N = 29,261,805 (55%) ^a^ | Yes, N = 23,847,875 (45%) ^a^ | No, N = 17,791,179 (33%) ^a^ | Yes, N = 35,318,502 (67%) ^a^ |
| Age^1234^ | 69.20 (6.65) | 69.78 (6.81) | 68.11 (6.20) | 69.56 (6.71) | 67.67 (6.15) | 69.74 (6.76) | 68.55 (6.45) | 70.46 (6.87) | 68.57 (6.45) |
| Age (Group)^1234^ |  |  |  |  |  |  |  |  |  |
| 60-69 years | 1,464 (51.72%) | 969 (47.75%) | 495 (59.29%) | 1,095 (49.10%) | 369 (62.92%) | 816 (48.88%) | 648 (55.21%) | 442 (43.40%) | 1,022 (55.91%) |
| 70-79 years | 806 (27.48%) | 561 (27.05%) | 245 (28.30%) | 672 (28.99%) | 134 (21.03%) | 479 (28.38%) | 327 (26.38%) | 298 (28.88%) | 508 (26.78%) |
| 80+ years | 654 (20.80%) | 515 (25.20%) | 139 (12.41%) | 547 (21.91%) | 107 (16.05%) | 411 (22.74%) | 243 (18.41%) | 291 (27.73%) | 363 (17.31%) |
| Sex^124^ |  |  |  |  |  |  |  |  |  |
| Female | 1,504 (54.54%) | 1,113 (57.50%) | 391 (48.91%) | 1,223 (56.29%) | 281 (47.09%) | 907 (56.55%) | 597 (52.07%) | 604 (61.02%) | 900 (51.27%) |
| Male | 1,420 (45.46%) | 932 (42.50%) | 488 (51.09%) | 1,091 (43.71%) | 329 (52.91%) | 799 (43.45%) | 621 (47.93%) | 427 (38.98%) | 993 (48.73%) |
| PIR^134^ | 3.12 (1.58) | 3.01 (1.60) | 3.32 (1.52) | 3.11 (1.58) | 3.16 (1.60) | 2.85 (1.58) | 3.44 (1.52) | 2.77 (1.58) | 3.29 (1.55) |
| Race^123^ |  |  |  |  |  |  |  |  |  |
| Non-Hispanic White | 1,396 (79.48%) | 924 (76.76%) | 472 (84.67%) | 1,170 (80.68%) | 226 (74.35%) | 820 (78.68%) | 576 (80.46%) | 510 (78.70%) | 886 (79.87%) |
| Non-Hispanic Black | 697 (8.44%) | 502 (9.41%) | 195 (6.60%) | 549 (8.42%) | 148 (8.53%) | 417 (9.14%) | 280 (7.59%) | 254 (9.43%) | 443 (7.94%) |
| Other Hispanic | 294 (3.66%) | 223 (4.26%) | 71 (2.50%) | 191 (3.02%) | 103 (6.37%) | 185 (4.22%) | 109 (2.97%) | 92 (3.62%) | 202 (3.67%) |
| Other/multiracial | 281 (5.04%) | 216 (5.93%) | 65 (3.34%) | 200 (4.57%) | 81 (7.02%) | 132 (4.28%) | 149 (5.97%) | 76 (4.27%) | 205 (5.42%) |
| Mexican American | 256 (3.39%) | 180 (3.64%) | 76 (2.90%) | 204 (3.31%) | 52 (3.73%) | 152 (3.69%) | 104 (3.02%) | 99 (3.97%) | 157 (3.09%) |
| Education attainment^1234^ |  |  |  |  |  |  |  |  |  |
| Less Than 9th Grade | 329 (5.70%) | 255 (6.97%) | 74 (3.28%) | 252 (5.74%) | 77 (5.56%) | 236 (7.96%) | 93 (2.94%) | 147 (9.06%) | 182 (4.01%) |
| 9-11th Grade | 415 (10.27%) | 313 (11.43%) | 102 (8.07%) | 316 (10.27%) | 99 (10.28%) | 300 (13.64%) | 115 (6.15%) | 178 (13.43%) | 237 (8.68%) |
| High School Grad/GED | 684 (22.19%) | 475 (22.21%) | 209 (22.15%) | 570 (23.74%) | 114 (15.56%) | 425 (24.32%) | 259 (19.57%) | 266 (25.88%) | 418 (20.33%) |
| Some College or AA degree | 821 (31.29%) | 523 (27.90%) | 298 (37.75%) | 649 (31.06%) | 172 (32.25%) | 460 (32.48%) | 361 (29.83%) | 252 (28.04%) | 569 (32.93%) |
| College Graduate or above | 672 (30.55%) | 476 (31.49%) | 196 (28.75%) | 526 (29.19%) | 146 (36.35%) | 283 (21.61%) | 389 (41.52%) | 188 (23.60%) | 484 (34.05%) |
| Drinking status^34^ |  |  |  |  |  |  |  |  |  |
| Non-drinker | 916 (27.35%) | 675 (28.81%) | 241 (24.59%) | 730 (28.08%) | 186 (24.24%) | 564 (31.97%) | 352 (21.71%) | 358 (33.08%) | 558 (24.49%) |
| 1-5 drinks/month | 1,379 (47.46%) | 949 (47.27%) | 430 (47.84%) | 1,101 (47.65%) | 278 (46.67%) | 822 (48.84%) | 557 (45.79%) | 490 (49.07%) | 889 (46.66%) |
| 5-10 drinks/month | 122 (5.00%) | 80 (4.20%) | 42 (6.50%) | 91 (4.28%) | 31 (8.03%) | 61 (3.87%) | 61 (6.37%) | 26 (2.43%) | 96 (6.28%) |
| 10+ drinks/month | 452 (20.19%) | 301 (19.72%) | 151 (21.08%) | 346 (19.98%) | 106 (21.06%) | 218 (15.33%) | 234 (26.13%) | 130 (15.42%) | 322 (22.57%) |
| Smoking status^3^ |  |  |  |  |  |  |  |  |  |
| Current smoker | 371 (10.96%) | 254 (10.61%) | 117 (11.61%) | 277 (10.63%) | 94 (12.36%) | 269 (14.69%) | 102 (6.37%) | 143 (13.03%) | 228 (9.91%) |
| Former smoker | 1,108 (39.33%) | 760 (37.91%) | 348 (42.02%) | 898 (39.67%) | 210 (37.86%) | 626 (36.87%) | 482 (42.35%) | 380 (36.47%) | 728 (40.77%) |
| Never smoker | 1,443 (49.72%) | 1,030 (51.47%) | 413 (46.36%) | 1,138 (49.70%) | 305 (49.77%) | 811 (48.44%) | 632 (51.28%) | 508 (50.50%) | 935 (49.32%) |
| BMI^234^ | 29.06 (6.29) | 29.28 (6.60) | 28.65 (5.64) | 29.31 (6.40) | 28.00 (5.70) | 29.95 (6.78) | 27.99 (5.46) | 30.48 (7.20) | 28.37 (5.67) |
| Trouble remembering^34^ |  |  |  |  |  |  |  |  |  |
| No | 1,626 (54.52%) | 1,152 (55.39%) | 474 (52.89%) | 1,280 (54.79%) | 346 (53.41%) | 955 (55.41%) | 671 (53.43%) | 565 (55.10%) | 1,061 (54.24%) |
| About once | 693 (26.43%) | 460 (25.73%) | 233 (27.76%) | 541 (26.18%) | 152 (27.49%) | 377 (23.95%) | 316 (29.47%) | 217 (23.58%) | 476 (27.86%) |
| Two or three times | 435 (14.06%) | 304 (13.83%) | 131 (14.50%) | 356 (14.27%) | 79 (13.18%) | 252 (14.33%) | 183 (13.72%) | 163 (14.32%) | 272 (13.93%) |
| Nearly every day | 116 (3.57%) | 85 (3.31%) | 31 (4.06%) | 93 (3.30%) | 23 (4.72%) | 81 (4.22%) | 35 (2.77%) | 56 (4.48%) | 60 (3.11%) |
| Several times a day | 50 (1.42%) | 40 (1.75%) | 10 (0.80%) | 41 (1.48%) | 9 (1.20%) | 38 (2.09%) | 12 (0.61%) | 27 (2.53%) | 23 (0.87%) |
| Depression^34^ |  |  |  |  |  |  |  |  |  |
| No | 2,598 (92.72%) | 1,801 (91.96%) | 797 (94.15%) | 2,046 (92.14%) | 552 (95.16%) | 1,463 (90.16%) | 1,135 (95.83%) | 868 (88.74%) | 1,730 (94.69%) |
| Yes | 261 (7.28%) | 196 (8.04%) | 65 (5.85%) | 214 (7.86%) | 47 (4.84%) | 195 (9.84%) | 66 (4.17%) | 132 (11.26%) | 129 (5.31%) |
| Hypertension^234^ |  |  |  |  |  |  |  |  |  |
| No | 954 (36.39%) | 640 (34.94%) | 314 (39.16%) | 706 (34.72%) | 248 (43.51%) | 513 (32.29%) | 441 (41.42%) | 277 (29.30%) | 677 (39.96%) |
| Yes | 1,970 (63.61%) | 1,405 (65.06%) | 565 (60.84%) | 1,608 (65.28%) | 362 (56.49%) | 1,193 (67.71%) | 777 (58.58%) | 754 (70.70%) | 1,216 (60.04%) |
| Diabetes^1234^ |  |  |  |  |  |  |  |  |  |
| No | 2,106 (76.49%) | 1,442 (74.36%) | 664 (80.55%) | 1,643 (75.50%) | 463 (80.74%) | 1,203 (73.43%) | 903 (80.24%) | 697 (70.71%) | 1,409 (79.40%) |
| Yes | 817 (23.51%) | 602 (25.64%) | 215 (19.45%) | 670 (24.50%) | 147 (19.26%) | 502 (26.57%) | 315 (19.76%) | 333 (29.29%) | 484 (20.60%) |
| Cardiovascular disease^234^ |  |  |  |  |  |  |  |  |  |
| No | 2,415 (82.40%) | 1,702 (83.37%) | 713 (80.54%) | 1,871 (80.71%) | 544 (89.61%) | 1,369 (80.21%) | 1,046 (85.09%) | 815 (78.80%) | 1,600 (84.21%) |
| Yes | 508 (17.60%) | 343 (16.63%) | 165 (19.46%) | 442 (19.29%) | 66 (10.39%) | 336 (19.79%) | 172 (14.91%) | 216 (21.20%) | 292 (15.79%) |
| CERAD.IR^134^ | 19.72 (4.49) | 19.47 (4.63) | 20.20 (4.17) | 19.65 (4.47) | 20.03 (4.58) | 19.20 (4.58) | 20.36 (4.30) | 18.97 (4.74) | 20.10 (4.31) |
| CERAD.DR^134^ | 6.23 (2.30) | 6.10 (2.40) | 6.48 (2.09) | 6.18 (2.32) | 6.43 (2.20) | 6.00 (2.34) | 6.51 (2.22) | 5.84 (2.45) | 6.43 (2.20) |
| Animal Fluency^1234^ | 18.08 (5.70) | 17.62 (5.87) | 18.95 (5.25) | 17.86 (5.66) | 19.00 (5.76) | 16.98 (5.45) | 19.44 (5.70) | 16.37 (5.30) | 18.94 (5.70) |
| DSST^1234^ | 51.96 (16.81) | 50.38 (17.54) | 54.98 (14.86) | 51.57 (16.84) | 53.62 (16.61) | 48.47 (17.10) | 56.24 (15.40) | 46.67 (17.35) | 54.63 (15.88) |
| Note: Unknown: PIR (248,8.5%); Depression (65,2.2%); Alcoholism (55,1.8%); BMI (49,1.6%); Smoking status (2); Diabetes (1); Cardiovascular disease (1); Trouble remembering (4) | | | | | | | | | |
| t-test adapted to complex survey samples and chi-squared test with Rao & Scott's second-order correction were used to calculate between-group differences. | | | | | | | | | |
| ^1234^ Represents the within-group differences of variables in people with and without OPA, TPA, RPA or total PA, respectively. | | | | | | | | | |
| ^a^ Mean (SD) for continuous; n (%) for categorical. | | | | | | | | | |

Supplementary Table 2. Subgroup analysis

Supplementary Table 2.1. CERAD.IR

|  | **CERAD.IR** | | | | | | | |
| --- | --- | --- | --- | --- | --- | --- | --- | --- |
|  | **Occupational PA** | | **Transport-related PA** | | **Recreational PA** | | **Total PA** | |
| Age (Group) | β (95% CI) | P-value | β (95% CI) | P-value | β (95% CI) | P-value | β (95% CI) | P-value |
| 60-69 years | -0.133(-0.940,0.673) | 0.708 | 0.286(-0.674, 1.246) | 0.504 | -0.389(-1.097,0.320) | 0.236 | -0.028(-0.590,0.534) | 0.909 |
| 70-79 years | 0.008(-0.771,0.787) | 0.982 | -0.359(-1.972, 1.253) | 0.615 | 0.496(-0.530, 1.521) | 0.291 | 0.427(-0.440, 1.294) | 0.282 |
| 80+ years | **1.061(0.002, 2.120)** | **0.050** | -0.476(-1.978, 1.027) | 0.479 | **1.021(0.170, 1.871)** | **0.025** | **1.050(0.469, 1.631)** | **0.004** |
| Sex |  |  |  |  |  |  |  |  |
| female | -0.015(-0.722,0.691) | 0.961 | -0.094(-1.165, 0.978) | 0.842 | 0.462(-0.157, 1.082) | 0.121 | 0.419(-0.156, 0.994) | 0.128 |
| male | 0.291(-0.598,1.180) | 0.464 | 0.054(-0.837, 0.945) | 0.891 | -0.235(-1.060,0.591) | 0.523 | 0.330(-0.430, 1.089) | 0.339 |
| Alcoholism |  |  |  |  |  |  |  |  |
| 1-5 drinks/month | -0.051(-0.658,0.557) | 0.854 | 0.303(-0.529, 1.136) | 0.431 | 0.333(-0.318, 0.984) | 0.277 | 0.489(-0.156, 1.134) | 0.121 |
| 5-10 drinks/month | -0.463(-2.947,2.022) | 0.595 | -1.479(-5.291, 2.334) | 0.305 | 0.072(-2.746, 2.890) | 0.940 | 0.635(-2.074, 3.344) | 0.510 |
| 10+ drinks/month | 0.136(-1.106,1.379) | 0.809 | -0.177(-1.484, 1.131) | 0.767 | -0.159(-1.320,1.003) | 0.764 | 0.325(-0.911, 1.562) | 0.566 |
| Non-drinker | 0.629(-0.244,1.502) | 0.138 | -0.365(-1.713, 0.984) | 0.556 | 0.038(-1.023, 1.099) | 0.937 | 0.155(-0.671, 0.981) | 0.681 |
| Smoking status |  |  |  |  |  |  |  |  |
| Current smoker | 0.256(-0.306, 0.819) | 0.324 | -0.059(-1.215, 1.097) | 0.909 | 0.098(-0.525, 0.721) | 0.726 | 0.214(-0.373, 0.802) | 0.425 |
| Former smoker | -0.177(-0.922, 0.568) | 0.598 | 0.174(-0.582, 0.929) | 0.610 | -0.045(-0.808,0.717) | 0.894 | 0.244(-0.384, 0.872) | 0.396 |
| Never smoker | 0.814(-0.652, 2.279) | 0.236 | -0.478(-2.247, 1.290) | 0.550 | **1.448(0.528, 2.368)** | **0.007** | **1.486(0.312, 2.661)** | **0.019** |
| Race |  |  |  |  |  |  |  |  |
| Non-Hispanic White | 0.084(-0.448, 0.617) | 0.731 | 0.102(-0.624, 0.828) | 0.760 | 0.068(-0.416, 0.553) | 0.760 | **0.413(0.008, 0.817)** | **0.046** |
| Other | 0.452(-0.031, 0.934) | 0.064 | -0.229(-1.119, 0.661) | 0.579 | 0.311(-0.226, 0.847) | 0.226 | 0.248(-0.262, 0.757) | 0.304 |
| Education attainment |  |  |  |  |  |  |  |  |
| Less Than 9th Grade | -0.032(-2.069, 2.004) | 0.972 | 0.057(-2.046, 2.159) | 0.952 | 0.368(-1.772, 2.509) | 0.702 | 0.087(-1.822, 1.997) | 0.919 |
| 9-11th Grade | 0.564(-0.289, 1.416) | 0.169 | -1.329(-3.547, 0.889) | 0.208 | 0.229(-1.471, 1.929) | 0.768 | 0.551(-0.595, 1.696) | 0.305 |
| High School Grad/GED | -0.139(-1.087, 0.809) | 0.748 | 0.157(-1.043, 1.357) | 0.774 | 0.141(-0.958, 1.240) | 0.778 | -0.030(-0.977,0.918) | 0.945 |
| Some College or AA degree | -0.113(-1.084, 0.859) | 0.801 | 0.250(-1.112, 1.612) | 0.691 | -0.104(-0.742,0.534) | 0.724 | **0.650(0.019, 1.281)** | **0.045** |
| College Graduate or above | 0.640(-0.422, 1.701) | 0.206 | -0.012(-1.015, 0.990) | 0.978 | 0.184(-0.741, 1.109) | 0.664 | 0.301(-0.467, 1.069) | 0.398 |

Based on the association between domain-specific PA and cognitive function in accordance with PA guidelines, a fully adjusted model was used to calculate the effect value (β) and 95% confidence interval (CI).

Significant values (P < 0.05) are in bold.

Supplementary Table 2.2. CERAD.DR

|  | **CERAD.DR** | | | | | | | |
| --- | --- | --- | --- | --- | --- | --- | --- | --- |
|  | **Occupational PA** | | **Transport-related PA** | | **Recreational PA** | | **Total PA** | |
| Age (Group) | β (95% CI) | P-value | β (95% CI) | P-value | β (95% CI) | P-value | β (95% CI) | P-value |
| 60-69 years | -0.118(-0.493, 0.257) | 0.480 | -0.025(-0.475, 0.424) | 0.897 | 0.006(-0.433, 0.444) | 0.977 | 0.025(-0.276, 0.327) | 0.848 |
| 70-79 years | -0.026(-0.497, 0.445) | 0.900 | 0.351(-0.479, 1.181) | 0.351 | 0.286(-0.314, 0.887) | 0.297 | 0.212(-0.270, 0.694) | 0.333 |
| 80+ years | **0.788(0.114, 1.463)** | **0.028** | 0.280(-0.563, 1.123) | 0.458 | **0.833(0.255, 1.412)** | **0.011** | **0.882(0.494, 1.270)** | **0.001** |
| Sex |  |  |  |  |  |  |  |  |
| female | 0.136(-0.231, 0.503 | 0.410 | 0.085(-0.421, 0.591) | 0.704 | 0.293(-0.023, 0.609) | 0.065 | **0.334(0.028, 0.641)** | **0.037** |
| male | -0.035(-0.440, 0.370) | 0.843 | -0.039(-0.504, 0.425) | 0.847 | 0.119(-0.422, 0.661) | 0.619 | 0.155(-0.228, 0.539) | 0.370 |
| Alcoholism |  |  |  |  |  |  |  |  |
| 1-5 drinks/month | -0.088(-0.377, 0.201) | 0.508 | 0.152(-0.378, 0.681) | 0.533 | 0.276(-0.143, 0.694) | 0.170 | 0.282(-0.067, 0.631) | 0.101 |
| 5-10 drinks/month | 0.044(-2.288, 2.375) | 0.956 | -1.121(-3.865, 1.622) | 0.284 | 0.661(-1.277, 2.598) | 0.357 | 0.973(-1.203, 3.149) | 0.250 |
| 10+ drinks/month | 0.063(-0.637, 0.762) | 0.844 | 0.345(-0.329, 1.019) | 0.277 | 0.236(-0.395, 0.867) | 0.420 | 0.257(-0.157, 0.670) | 0.194 |
| Non-drinker | 0.287(-0.141, 0.715) | 0.164 | -0.060(-0.805, 0.685) | 0.859 | 0.058(-0.486, 0.602) | 0.814 | 0.129(-0.317, 0.576) | 0.528 |
| Smoking status |  |  |  |  |  |  |  |  |
| Current smoker | -0.040(-0.335, 0.255) | 0.762 | -0.073(-0.721, 0.575) | 0.802 | 0.179(-0.147, 0.504) | 0.241 | 0.125(-0.194, 0.444) | 0.394 |
| Former smoker | 0.071(-0.331, 0.472) | 0.696 | 0.145(-0.481, 0.771) | 0.608 | 0.146(-0.266, 0.558) | 0.438 | 0.250(-0.113, 0.614) | 0.151 |
| Never smoker | 0.396(-0.320, 1.112) | 0.238 | 0.331(-0.700, 1.361) | 0.481 | **0.910(0.330, 1.491)** | **0.007** | **0.759(0.135, 1.383)** | **0.023** |
| Race |  |  |  |  |  |  |  |  |
| Non-Hispanic White | 0.028(-0.224, 0.281) | 0.807 | 0.078(-0.368, 0.523) | 0.705 | 0.220(-0.054, 0.495) | 0.104 | **0.296(0.101, 0.492)** | **0.007** |
| Other | 0.173(-0.130, 0.476) | 0.232 | -0.005(-0.482, 0.472) | 0.984 | 0.249(-0.092, 0.589) | 0.134 | 0.155(-0.140, 0.449) | 0.270 |
| Education attainment |  |  |  |  |  |  |  |  |
| Less Than 9th Grade | 0.108(-0.801, 1.016) | 0.791 | 0.088(-0.950, 1.126) | 0.850 | 0.355(-0.599, 1.308) | 0.416 | 0.214(-0.700, 1.129) | 0.604 |
| 9-11th Grade | 0.344(-0.042, 0.730) | 0.075 | 0.079(-1.182, 1.340) | 0.890 | 0.218(-0.573, 1.009) | 0.549 | 0.334(-0.206, 0.874) | 0.196 |
| High School Grad/GED | 0.030(-0.520, 0.579) | 0.906 | 0.616(-0.265, 1.497) | 0.148 | 0.116(-0.435, 0.667) | 0.646 | 0.086(-0.339, 0.511) | 0.658 |
| Some College or AA degree | -0.046(-0.544, 0.452) | 0.841 | -0.197(-0.801, 0.407) | 0.483 | -0.014(-0.498, 0.469) | 0.948 | 0.254(-0.183, 0.691) | 0.225 |
| College Graduate or above | 0.058(-0.417, 0.534) | 0.788 | -0.026(-0.616, 0.564) | 0.922 | 0.443(-0.075, 0.962) | 0.085 | 0.303(-0.207, 0.813) | 0.212 |

Based on the association between domain-specific PA and cognitive function in accordance with PA guidelines, a fully adjusted model was used to calculate the effect value (β) and 95% confidence interval (CI).

Significant values (P < 0.05) are in bold.

Supplementary Table 2.3. Animal Fluency test（AF）

|  | **Animal Fluency test（AF）** | | | | | | | |
| --- | --- | --- | --- | --- | --- | --- | --- | --- |
|  | **Occupational PA** | | **Transport-related PA** | | **Recreational PA** | | **Total PA** | |
| Age (Group) | β (95% CI) | P-value | β (95% CI) | P-value | β (95% CI) | P-value | β (95% CI) | P-value |
| 60-69 years | -0.480(-1.200, 0.240) | 0.159 | -0.356(-1.529, 0.816) | 0.496 | **0.928(0.057, 1.798)** | **0.040** | 0.364(-0.381, 1.110) | 0.297 |
| 70-79 years | 0.387(-0.846, 1.621) | 0.482 | **2.018(0.574, 3.462)** | **0.013** | **1.970(0.763, 3.177)** | **0.006** | **1.872(0.822, 2.921)** | **0.003** |
| 80+ years | 1.101(-0.242, 2.444) | 0.094 | 1.911(-1.010, 4.832) | 0.166 | **1.997(0.811, 3.184)** | **0.005** | **1.944(1.089, 2.799)** | **<0.001** |
| Sex |  |  |  |  |  |  |  |  |
| female | 0.328(-0.406, 1.062) | 0.326 | 0.522(-0.970, 2.014) | 0.435 | **1.605(0.765, 2.446)** | **0.003** | **1.260(0.630, 1.889)** | **0.001** |
| male | -0.112(-1.193, 0.968) | 0.813 | 0.399(-0.828, 1.627) | 0.467 | **1.225(0.215, 2.236)** | **0.024** | **0.989(0.011, 1.966)** | **0.048** |
| Alcoholism |  |  |  |  |  |  |  |  |
| 1-5 drinks/month | 0.050(-0.790, 0.891) | 0.895 | 1.106(-0.344, 2.557) | 0.119 | **1.632(0.679, 2.584)** | **0.004** | **1.478(0.815, 2.141)** | **<0.001** |
| 5-10 drinks/month | 0.706(-2.534, 3.946) | 0.538 | -1.008(-7.513, 5.496) | 0.656 | 0.937(-4.367, 6.241) | 0.613 | **3.691(0.154, 7.229)** | **0.044** |
| 10+ drinks/month | -0.976(-2.779, 0.828) | 0.252 | 0.871(-0.979, 2.722) | 0.315 | **1.445(0.121, 2.769)** | **0.036** | 0.469(-0.691, 1.630) | 0.392 |
| Non-drinker | 0.911(-0.008, 1.829) | 0.052 | -0.393(-1.548, 0.762) | 0.462 | 0.965(-0.307, 2.237) | 0.120 | 0.822(-0.024, 1.668) | 0.056 |
| Smoking status |  |  |  |  |  |  |  |  |
| Current smoker | 0.283(-0.491, 1.058) | 0.423 | 0.361(-1.013, 1.735) | 0.562 | **1.491(0.745, 2.237)** | **0.002** | **1.034(0.256, 1.812)** | **0.015** |
| Former smoker | -0.271(-1.294, 0.752) | 0.558 | 0.722(-0.725, 2.169) | 0.283 | **1.260(0.354, 2.167)** | **0.013** | **1.225(0.283, 2.166)** | **0.017** |
| Never smoker | 0.841(-0.424, 2.107) | 0.164 | -1.139(-3.403, 1.125) | 0.280 | **2.027(0.388, 3.665)** | **0.021** | **1.680(0.389, 2.972)** | **0.017** |
| Race |  |  |  |  |  |  |  |  |
| Non-Hispanic White | -0.113(-0.641, 0.415) | 0.644 | 0.363(-0.985, 1.710) | 0.562 | **1.571(0.906, 2.237)** | **<0.001** | **1.312(0.648, 1.976)** | **<0.001** |
| Other | 0.617(-0.135, 1.369) | 0.098 | 0.399(-0.414, 1.212) | 0.3 | 0.634(-0.108, 1.377) | 0.086 | **0.554(0.115, 0.994)** | **0.017** |
| Education attainment |  |  |  |  |  |  |  |  |
| Less Than 9th Grade | 0.614(-1.798, 3.027) | 0.573 | -0.153(-1.908, 1.602) | 0.846 | 0.403(-1.566, 2.372) | 0.650 | 0.427(-1.079, 1.934) | 0.541 |
| 9-11th Grade | 0.449(-1.780, 2.679) | 0.659 | -0.128(-1.470, 1.214) | 0.834 | 0.829(-0.646, 2.303) | 0.235 | 1.146(-0.144, 2.436) | 0.076 |
| High School Grad/GED | 0.414(-0.689, 1.516) | 0.418 | 0.813(-1.040, 2.667) | 0.347 | **1.402(0.092, 2.713)** | **0.039** | 0.515(-0.563, 1.594) | 0.315 |
| Some College or AA degree | 0.261(-0.828, 1.349) | 0.605 | 0.891(-0.624, 2.406) | 0.219 | **0.847(0.009, 1.686)** | **0.048** | **0.991(0.105, 1.877)** | **0.031** |
| College Graduate or above | -0.616(-1.557, 0.325) | 0.173 | -0.454(-2.420, 1.513) | 0.614 | **2.035(0.865, 3.205)** | **0.003** | **1.568(0.529, 2.607)** | **0.007** |

Based on the association between domain-specific PA and cognitive function in accordance with PA guidelines, a fully adjusted model was used to calculate the effect value (β) and 95% confidence interval (CI).

Significant values (P < 0.05) are in bold.

Supplementary Table 2.4. Digit Symbol Substitution test (DSST)

|  | **Digit Symbol Substitution test (DSST)** | | | | | | | |
| --- | --- | --- | --- | --- | --- | --- | --- | --- |
|  | **Occupational PA** | | **Transport-related PA** | | **Recreational PA** | | **Total PA** | |
| Age (Group) | β (95% CI) | P-value | β (95% CI) | P-value | β (95% CI) | P-value | β (95% CI) | P-value |
| 60-69 years | -0.069(-2.749, 2.611) | 0.953 | -1.447(-3.917, 1.023) | 0.208 | 1.839(-0.424, 4.101) | 0.096 | **2.540(0.744, 4.335)** | **0.011** |
| 70-79 years | 1.507(-1.260, 4.275) | 0.239 | -0.464(-4.309, 3.381) | 0.784 | 1.800(-0.599, 4.198) | 0.119 | **5.123(2.595, 7.651)** | **0.001** |
| 80+ years | 2.104(-2.660, 6.869) | 0.331 | 1.054(-4.304, 6.412) | 0.656 | **6.267(2.956, 9.579)** | **0.003** | **6.233(2.921, 9.545)** | **0.003** |
| Sex |  |  |  |  |  |  |  |  |
| female | 1.608(-1.073, 4.289) | 0.199 | -1.300(-4.709, 2.110) | 0.397 | 2.373(-0.099, 4.844) | 0.057 | **3.305(1.599, 5.012)** | **0.002** |
| male | 0.282(-2.308, 2.873) | 0.804 | -0.636(-3.215, 1.943) | 0.578 | **3.300(1.257, 5.343)** | **0.007** | **2.698(0.679, 4.717)** | **0.014** |
| Alcoholism |  |  |  |  |  |  |  |  |
| 1-5 drinks/month | 0.750 -1.607, 3.107) | 0.490 | -1.350(-4.337, 1.637) | 0.333 | **2.855(0.736, 4.975)** | **0.014** | **3.269(1.630, 4.908)** | **0.001** |
| 5-10 drinks/month | 2.508(-2.933, 7.948) | 0.239 | -5.660(-16.125, 4.806) | 0.184 | 0.159(-6.673, 6.990) | 0.946 | 3.148(-3.410, 9.706) | 0.272 |
| 10+ drinks/month | -0.634(-4.496, 3.227) | 0.719 | 1.618(-1.925, 5.160) | 0.329 | **2.933(0.149, 5.717)** | **0.041** | **3.563(1.020, 6.106)** | **0.010** |
| Non-drinker | 1.241(-1.601, 4.084) | 0.349 | -1.753(-5.400, 1.894) | 0.305 | 1.969(-1.189, 5.126) | 0.192 | 2.097(-0.208, 4.402) | 0.071 |
| Smoking status |  |  |  |  |  |  |  |  |
| Current smoker | 0.451(-2.048, 2.951) | 0.668 | -1.411(-4.238, 1.415) | 0.283 | **2.905(0.836, 4.973)** | **0.012** | **3.030(1.274, 4.786)** | **0.004** |
| Former smoker | 0.781(-1.849, 3.410) | 0.513 | -0.002(-3.797, 3.793) | 0.999 | **2.834(0.558, 5.109)** | **0.021** | **3.209(1.057, 5.361)** | **0.009** |
| Never smoker | 2.580(-1.736, 6.896) | 0.205 | -3.085(-8.588, 2.418) | 0.232 | 0.236(-4.122, 4.593) | 0.904 | 2.114(-0.786, 5.015) | 0.131 |
| Race |  |  |  |  |  |  |  |  |
| Non-Hispanic White | 0.643(-1.605, 2.890) | 0.538 | -1.558(-4.140, 1.024) | 0.208 | **3.333(1.465, 5.200)** | **0.003** | **3.691(2.078, 5.305)** | **<0.001** |
| Other | 0.888(-1.720, 3.496) | 0.466 | 1.096(-1.891, 4.083) | 0.433 | 2.032(-0.269, 4.334) | 0.077 | 1.211(-0.716, 3.138) | 0.199 |
| Education attainment |  |  |  |  |  |  |  |  |
| Less Than 9th Grade | -0.460(-4.663, 3.743) | 0.807 | -0.382(-5.335, 4.572) | 0.863 | 3.691(-0.374, 7.757) | 0.07 | -0.168(-3.299, 2.963) | 0.907 |
| 9-11th Grade | 1.744(-1.444, 4.932) | 0.247 | -1.260(-4.981, 2.462) | 0.463 | 4.276(-0.713, 9.266) | 0.084 | **3.125(0.039, 6.210)** | **0.048** |
| High School Grad/GED | 1.678(-1.693, 5.048) | 0.289 | 0.955(-3.279, 5.188) | 0.622 | 1.553(-2.258, 5.363) | 0.381 | 2.329(-0.505, 5.164) | 0.098 |
| Some College or AA degree | 0.132(-3.140, 3.403) | 0.93 | -2.780(-6.058, 0.498) | 0.088 | 1.015(-1.391, 3.421) | 0.369 | 2.247(-0.168, 4.663) | 0.065 |
| College Graduate or above | 0.453(-2.651, 3.557) | 0.749 | -0.437(-3.368, 2.493) | 0.743 | **4.690(2.048, 7.332)** | **0.003** | -0.168(-3.299, 2.963) | 0.907 |

Based on the association between domain-specific PA and cognitive function in accordance with PA guidelines, a fully adjusted model was used to calculate the effect value (β) and 95% confidence interval (CI).

Significant values (P < 0.05) are in bold.

Supplementary Table 3. Dose-response relationships between domain-specific PA and cognitive function

Supplementary Table 3.1. Dose-response relationships between domain-specific PA and cognitive function (based on model 1)

|  | **CERAD.IR** | | **CERAD.DR** | | **Animal. Fluency** | | **DSST** | |
| --- | --- | --- | --- | --- | --- | --- | --- | --- |
|  | β (95% CI) | p-value | β (95% CI) | p-value | β (95% CI) | p-value | β (95% CI) | p-value |
| **OPA** |  |  |  |  |  |  |  |  |
| None | reference |  | reference |  | reference |  | reference |  |
| Low | **0.846(0.211, 1.482)** | **0.011** | **0.482(0.097, 0.867)** | **0.016** | **1.741(0.809, 2.673)** | **<0.001** | **5.016(2.482, 7.551)** | **<0.001** |
| Moderate | **1.084(0.423, 1.745)** | **0.002** | **0.459(0.054, 0.864)** | **0.028** | **1.147(0.102, 2.192)** | **0.033** | **5.485(0.871, 10.100)** | **0.021** |
| High | 0.567(-0.106, 1.241) | 0.096 | **0.288(0.035, 0.541)** | **0.027** | **1.156(0.318, 1.993)** | **0.009** | **4.112(1.208, 7.016)** | **0.007** |
|  | p for trend | 0.137 | p for trend | **0.047** | p for trend | **0.013** | p for trend | **0.010** |
| **TPA** |  |  |  |  |  |  |  |  |
| None | reference |  | reference |  | reference |  | reference |  |
| Low | 0.532(-0.342, 1.406) | 0.223 | 0.310(-0.146, 0.765) | 0.175 | **1.171(0.115, 2.227)** | **0.031** | **3.738(0.251, 7.225)** | **0.037** |
| Moderate | 0.393(-0.263, 1.050) | 0.230 | 0.280(-0.214, 0.773) | 0.256 | **1.680(0.122, 3.237)** | **0.035** | 2.210(-0.824, 5.244) | 0.147 |
| High | 0.155(-0.952, 1.261) | 0.777 | 0.137(-0.467, 0.741) | 0.647 | 0.480(-0.654, 1.614) | 0.394 | -0.344(-3.727, 3.038) | 0.837 |
|  | p for trend | 0.590 | p for trend | 0.418 | p for trend | 0.115 | p for trend | 0.750 |
| **RPA** |  |  |  |  |  |  |  |  |
| None | reference |  | reference |  | reference |  | reference |  |
| Low | **1.219(0.531, 1.908)** | **0.001** | **0.376(0.027, 0.725)** | **0.036** | **1.390(0.743, 2.038)** | **<0.001** | **5.701(3.545, 7.857)** | **<0.001** |
| Moderate | **1.148(0.606, 1.690)** | **<0.001** | **0.555(0.279, 0.831)** | **<0.001** | **2.548(1.621, 3.475)** | **<0.001** | **8.358(5.370, 11.347)** | **<0.001** |
| High | **1.125(0.590, 1.661)** | **<0.001** | **0.603(0.304, 0.902)** | **<0.001** | **3.417(2.566, 4.267)** | **<0.001** | **9.238(6.814, 11.662)** | **<0.001** |
|  | p for trend | **<0.001** | p for trend | **<0.001** | p for trend | **<0.001** | p for trend | **<0.001** |
| **Total PA** |  |  |  |  |  |  |  |  |
| None | reference |  | reference |  | reference |  | reference |  |
| Low | 0.773(-0.043, 1.588) | 0.062 | 0.378(-0.030, 0.785) | 0.068 | **1.739(1.052, 2.426)** | **<0.001** | **5.278(3.248, 7.308)** | **<0.001** |
| Moderate | **1.046(0.495, 1.596)** | **<0.001** | **0.474(0.189, 0.759)** | **0.002** | **2.403(1.460, 3.346)** | **<0.001** | **8.072(5.569, 10.575)** | **<0.001** |
| High | **1.311(0.924, 1.698)** | **<0.001** | **0.712(0.476, 0.947)** | **<0.001** | **2.977(2.298, 3.657)** | **<0.001** | **9.027(6.989, 11.065)** | **<0.001** |
|  | p for trend | **<0.001** | p for trend | **<0.001** | p for trend | **<0.001** | p for trend | **<0.001** |

Supplementary Table 3.2. Dose-response relationships between domain-specific PA and cognitive function (based on model 2)

|  | **CERAD.IR** | | **CERAD.DR** | | **Animal. Fluency** | | **DSST** | |
| --- | --- | --- | --- | --- | --- | --- | --- | --- |
|  | β (95% CI) | p-value | β (95% CI) | p-value | β (95% CI) | p-value | β (95% CI) | p-value |
| **OPA** |  |  |  |  |  |  |  |  |
| None | reference |  | reference |  | reference |  | reference |  |
| Low | 0.454(-0.119, 1.027) | 0.110 | 0.317(-0.036, 0.670) | 0.074 | **1.173(0.090, 2.257)** | **0.036** | **2.380(0.130, 4.630)** | **0.040** |
| Moderate | **0.640(0.048, 1.232)** | **0.036** | 0.270(-0.197, 0.737) | 0.232 | 0.232(-0.422, 0.885) | 0.460 | 2.420(-0.256, 5.095) | 0.073 |
| High | 0.057(-0.521, 0.635) | 0.834 | 0.041(-0.220, 0.302) | 0.737 | 0.261(-0.472, 0.993) | 0.158 | 0.813(-1.284, 2.910) | 0.420 |
|  | p for trend | 0.987 | p for trend | 0.961 | p for trend | 0.664 | p for trend | 0.575 |
| **TPA** |  |  |  |  |  |  |  |  |
| None | reference |  | reference |  | reference |  | reference |  |
| Low | 0.255(-0.517, 1.027) | 0.485 | 0.178(-0.195, 0.550) | 0.319 | 0.536(-0.328, 1.401) | 0.205 | **2.542(0.497, 4.587)** | **0.018** |
| Moderate | -0.027(-0.656, 0.602) | 0.926 | 0.043(-0.455, 0.541) | 0.854 | 0.943(-0.452, 2.338) | 0.169 | -0.434(-3.099, 2.232) | 0.732 |
| High | 0.094(-0.787, 0.974) | 0.821 | 0.077(-0.439, 0.592) | 0.752 | -0.042(-0.970, 0.887) | 0.924 | -0.926(-3.089, 1.237) | 0.374 |
|  | p for trend | 0.840 | p for trend | 0.701 | p for trend | 0.630 | p for trend | 0.362 |
| **RPA** |  |  |  |  |  |  |  |  |
| None | reference |  | reference |  | reference |  | reference |  |
| Low | 0.602(-0.040, 1.243) | 0.063 | 0.134(-0.214, 0.482) | 0.417 | 0.580(-0.061, 1.221) | 0.073 | **2.235(0.693, 3.776)** | **0.008** |
| Moderate | 0.468(-0.141, 1.077) | 0.120 | 0.305(-0.029, 0.639) | 0.070 | **1.462(0.663, 2.262)** | **0.002** | **4.099(2.205, 5.993)** | **<0.001** |
| High | 0.256(-0.151, 0.663) | 0.196 | **0.273(0.007, 0.538)** | **0.045** | **1.839(1.116, 2.562)** | **<0.001** | **3.509(1.454, 5.565)** | **0.003** |
|  | p for trend | 0.197 | p for trend | **0.027** | p for trend | **<0.001** | p for trend | **0.001** |
| **Total PA** |  |  |  |  |  |  |  |  |
| None | reference |  | reference |  | reference |  | reference |  |
| Low | 0.328(-0.339, 0.994) | 0.305 | **0.199(-0.203, 0.602)** | 0.302 | **1.107(0.437, 1.776)** | **0.003** | **2.801(1.078, 4.525)** | **0.004** |
| Moderate | **0.668(0.128, 1.209)** | **0.020** | **0.326(0.054, 0.597)** | **0.023** | **1.700(0.742, 2.658)** | **0.002** | **5.235(3.370, 7.101)** | **<0.001** |
| High | **0.503(0.087, 0.918)** | **0.022** | **0.373(0.101, 0.645)** | **0.011** | **1.543(0.888, 2.199)** | **<0.001** | **3.859(2.336, 5.382)** | **<0.001** |
|  | p for trend | **0.055** | p for trend | **0.015** | p for trend | **<0.001** | p for trend | **<0.001** |

Supplementary Table 4. Sensitivity analysis

|  | **CERAD.IR** | | **CERAD.DR** | | **AF** | | **DSST** | |
| --- | --- | --- | --- | --- | --- | --- | --- | --- |
| **Occupational PA** | β (95% CI) | P-value | β (95% CI) | P-value | β (95% CI) | P-value | β (95% CI) | P-value |
| NO | reference |  | reference |  | reference |  | reference |  |
| YES | 0.125(-0.435, 0.685) | 0.569 | 0.040(-0.231, 0.311) | 0.704 | -0.003(-0.547, 0.541) | 0.989 | 0.728(-1.641, 3.096) | 0.442 |
| None | reference |  | reference |  | reference |  | reference |  |
| Low | 0.431(-0.647, 1.510) | 0.227 | 0.323(-0.361, 1.006) | 0.179 | 1.168(-0.874, 3.210) | 0.133 | 2.538(-1.820, 6.896) | 0.129 |
| Moderate | 0.633(-0.520, 1.785) | 0.142 | 0.277(-0.619, 1.172) | 0.315 | 0.073(-1.332, 1.478) | 0.844 | 2.102(-3.177, 7.381) | 0.229 |
| High | 0.051(-0.997, 1.099) | 0.855 | 0.029(-0.460, 0.517) | 0.824 | 0.200(-1.093, 1.492) | 0.575 | 0.791(-3.217, 4.800) | 0.485 |
|  | p for trend | 0.999 | p for trend | 0.941 | p for trend | 0.596 | p for trend | 0.611 |
| **Transport-related PA** | β (95% CI) | P-value | β (95% CI) | P-value | β (95% CI) | P-value | β (95% CI) | P-value |
| NO | reference |  | reference |  | reference |  | reference |  |
| YES | -0.021(-0.758, 0.716) | 0.94 | 0.023(-0.408, 0.455) | 0.888 | 0.211(-1.080, 1.502) | 0.674 | -1.470(-3.844, 0.903) | 0.161 |
| None | reference |  | reference |  | reference |  | reference |  |
| Low | 0.240(-1.164, 1.645) | 0.538 | 0.166(-0.588, 0.920) | 0.443 | 0.400(-1.191, 1.992) | 0.392 | 2.175(-1.917, 6.268) | 0.149 |
| Moderate | -0.034(-1.426, 1.359) | 0.926 | 0.042(-0.954, 1.037) | 0.874 | 0.784(-1.934, 3.502) | 0.34 | -0.839(-6.057, 4.380) | 0.561 |
| High | 0.046(-1.558, 1.649) | 0.914 | 0.037(-0.945, 1.019) | 0.886 | -0.389(-2.287, 1.509) | 0.471 | -1.761(-6.833, 3.311) | 0.274 |
|  | p for trend | 0.925 | p for trend | 0.852 | p for trend | 0.837 | p for trend | 0.191 |
| **Recreational PA** | β (95% CI) | P-value | β (95% CI) | P-value | β (95% CI) | P-value | β (95% CI) | P-value |
| NO | reference |  | reference |  | reference |  | reference |  |
| YES | 0.111(-0.428, 0.650) | 0.597 | 0.214(-0.084, 0.513) | 0.117 | **1.383(0.744, 2.021)** | **0.003** | **2.595(0.943, 4.247)** | **0.01** |
| None | reference |  | reference |  | reference |  | reference |  |
| Low | 0.511(-0.755, 1.777) | 0.225 | 0.097(-0.592, 0.785) | 0.607 | 0.495(-0.479, 1.469) | 0.204 | 1.960(-0.299, 4.220) | 0.07 |
| Moderate | 0.333(-0.913, 1.578) | 0.369 | 0.253(-0.423, 0.929) | 0.249 | **1.308(0.110, 2.506)** | **0.04** | **3.354(0.776, 5.931)** | **0.026** |
| High | 0.153(-0.634, 0.939) | 0.492 | 0.225(-0.317, 0.768) | 0.216 | **1.705(0.670, 2.740)** | **0.013** | **2.847(-0.093, 5.786)** | **0.054** |
|  | p for trend | 0.497 | p for trend | 0.121 | p for trend | **0.004** | p for trend | **0.026** |

Sensitivity analysis by adding the timing of PA in the other two domains in the fully adjusted model.

Significant values (P < 0.05) are in bold
